# Becoming an Agile Change Conductor

**DOI:** 10.3389/fpubh.2022.1044702

**Published:** 2022-12-14

**Authors:** Jade Mehta, Matthew C. Aalsma, Andrew O'Brien, Tanna J. Boyer, Rami A. Ahmed, Diana Summanwar, Malaz Boustani

**Affiliations:** ^1^Center for Health Innovation and Implementation Science, School of Medicine, Indiana University, Indianapolis, IN, United States; ^2^Department of Pediatrics, School of Medicine, Indiana University, Indianapolis, IN, United States; ^3^Department of Medicine, School of Medicine, Indiana University, Indianapolis, IN, United States; ^4^Department of Anesthesia, School of Medicine, Indiana University, Indianapolis, IN, United States; ^5^Division of Simulation, Department of Emergency Medicine, Indiana University School of Medicine, Indianapolis, IN, United States; ^6^Department of Family Medicine, School of Medicine, Indiana University, Indianapolis, IN, United States; ^7^Center for Aging Research, Regenstrief Institute, Inc., Indianapolis, IN, United States; ^8^Sandra Eskenazi Center for Brain Care Innovation, Eskenazi Health, Indianapolis, IN, United States

**Keywords:** innovation, diffusion, quality improvement, educational review, implementation science, Agile

## Abstract

**Background:**

It takes decades and millions of dollars for a new scientific discovery to become part of clinical practice. In 2015, the Center for Health Innovation & Implementation Science (CHIIS) launched a Professional Certificate Program in Innovation and Implementation Sciences aimed at transforming healthcare professionals into Agile Change Conductors capable of designing, implementing, and diffusing evidence-based healthcare solutions.

**Method:**

In 2022, the authors surveyed alumni from the 2016–2021 cohorts of the Certificate Program as part of an educational quality improvement inquiry and to evaluate the effectiveness of the program.

**Results:**

Of the 60 alumni contacted, 52 completed the survey (87% response rate) with 60% of graduates being female while 30% were an under-represented minority. On a scale from 1 to 5, the graduates agreed that the certificate benefited their careers (4.308 with a standard deviation (SD) of 0.612); expanded their professional network (4.615, SD of 0.530); and had a large impact on the effectiveness of their leadership (4.288, SD of 0.667), their change management (4.365, SD of 0.742), and their communication (4.392, SD of 0.666). Graduates claimed to use Agile Processes (Innovation, Implementation, or Diffusion), storytelling, and nudging weekly. On a scale from 0 to 10 where 10 indicates reaching a mastery, the average score for different Agile competencies ranged from 5.37 (SD of 2.80) for drafting business proposals to 7.77 (SD of 1.96) for self-awareness. For the 2020 and 2021 cohorts with existing pre and post training competency data, 22 of the 26 competencies saw a statistically significant increase.

**Conclusion:**

The Graduate Certificate has been able to create a network of Agile Change Conductors competent to design, implement, and diffuse evidence-based care within the healthcare delivery system. Further improvements in building dissemination mastery and program expansion initiatives are advised.

## Introduction

In United States healthcare delivery organizations, 40% of patients do not receive evidence-based care, ~25% of patients are harmed, and at least 30% of annual healthcare spending is wasted (1–3). Harm events include medication errors, injuries, infection, prolonged hospitalization, or other unintended consequences (2). At the same time, the scientific community publishes more than 140 clinical trials and 80 systematic evidence reviews every single day (4, 5). Still, it takes decades and millions of dollars for a new scientific discovery to become part of clinical practice (6–9). For example, over the past two decades numerous randomized control trials have demonstrated the effectiveness of collaborative care models for depression and dementia. Nevertheless, the probability of patients receiving these evidence-based models is very low (10–13). Determinants that prevent the adoption of evidence-based practices include inconsistent internal demands, stakeholder resistance, unstable leadership, and inadequate resources (14).

To address the gap between knowledge and practice, healthcare delivery organizations must acquire workforces with competency in designing, implementing, and diffusing evidence-based healthcare solutions. In 2015, the Indiana University Center for Health Innovation & Implementation Science (CHIIS) launched a Professional Certificate Program in Innovation and Implementation Sciences as an attempt to build a network of Agile Change Conductors. This graduate educational program sought to train established healthcare professionals, consisting of clinicians, educators, researchers, and administrators, to be able to leverage insights from Agile Science to design, implement, scale, sustain, and diffuse evidence-based care solutions within their local complex adaptive healthcare delivery organizations (15). Derived from the root of the word “Agile”, which refers to speed and adaptability, Agile Science is an evolving and adaptive discovery and acquisition process for understanding, predicting, and steering the behavior of an individual human or the behavior of a social human organization, such as a healthcare delivery organization (16–18). The program seeks to equip clinicians and healthcare leaders with tools, processes, and strategies to create demand, encourage positive behaviors, foster a reinforcing culture, and address setbacks quickly and with precision to create multidisciplinary systemic change.

The purpose of this paper is to describe and evaluate the effectiveness of the Graduate Certificate from its inception in the Fall of 2015 until the Summer of 2021 and discuss areas for quality improvement.

## Methods

### Study design

A survey of all alumni of the Certificate Program in Innovation and Implementation Sciences (IIS) was conducted to evaluate program effectiveness and as part of an educational quality improvement inquiry. The complete survey is available as Supplementary Appendix 1. This project was approved by the Indiana University Institutional Review Board (#15824).

### Structure of the (IIS) Certificate Program

The Certificate Program is offered as a graduate course through the Indiana University School of Medicine. The course is 12 months long with 4 hour-long video meetings and one weekend residency each month (19). From 2015 to 2019, the program included a mix of in-person and virtual learning sessions; with weekly evening lectures and discussions in the form of online video conferences (Zoom) and monthly residencies in-person. In-person residencies took place at the Indiana University Health Information and Translational Sciences building in Indianapolis, IN. Since the COVID-19 pandemic, the 2020 and beyond cohorts have virtually completed the program. Full attendance provides 240 h of virtual or in-person meetings. Coursework in the form of readings, extra practice opportunities, and assignments must be completed outside of the 240 h. Full completion awards 15 credit hours through Indiana University, with a cost of $1,323.50 per credit hour (18). The program is taught by Dr. Malaz Boustani, MD, MPH, Richard M. Fairbanks Professor of Aging Research, Founding Director of the Center for Health Innovation and Implementation Science. To apply, applications must be submitted through the Indiana University Graduate School application portal (19). Requirements for the program are a bachelor's degree, 2 years of experience in the healthcare field, a curriculum vitae, and a written statement of interest. The program is intended for physicians, nurses, administrators, executives, researchers, and other leaders in healthcare passionate about being effective change agents within their local complex adaptive social organization.

The weekly 1-h coaching sessions focus on real-world deliberate practice and open-space discussions. The format of Saturday weekend residencies: group reflection 8:00–10:00 a.m., guest speaker 10:00 a.m.−12:00 p.m., lunch break, guest speaker 1:00–3:00 p.m., group reflection 3:00–5:00 p.m. Speakers have ranged from CEOs to business founders to alumni of the program. During the allocated time for Agile Reflection, participants are asked to rate their experience from −10 and +10 and share two English words, what they found interesting, surprising, actionable, and anything worth converting into an artifact, nudge, or ritual (see Supplementary Appendix 2). Sunday residencies are similarly from 8:00 a.m. to 5:00 p.m. with an hour break for lunch and consist of presentations and lectures by faculty focused on knowledge acquisition, application of topics, or structured work time. Additional projects may be completed outside of these designated class hours.

The Certificate Program concentrates on knowledge acquisition through readings and lectures and demonstration of knowledge acquisition through deliberate practice coupled with personal and group feedback. The intention of the small group size, 16 students or less, is to foster strong interpersonal connections and create time for individual mentoring built on a foundation of psychological safety. To maximize learning, there must be enough trust to check assumptions and ask questions or voice concerns. In doing so, the sessions become one of collaboration and critical thinking rather than passive learning. This is done by building time and space for reflection as well as timely, actionable, and non-judgmental feedback. Students are encouraged to share when they apply Agile Science in their personal and professional spheres, creating group learning on how to fail quickly and providing examples detailing attributes of successful implementation.

The Certificate Program is pass or fail, graded by weekly participation (25%), residency participation (25%), and the final project (50%). The first major assignment is to describe oneself as a company, detailing the tagline, mission, vision, and values. This is converted into a minimally viable story, used by students to introduce themselves to guest speakers, and can serve as a blueprint for branding. Students are encouraged to reach out to speakers after their presentations using LinkedIn™ to network for discovery. During the first 4 months of the certificate, students are encouraged to find ways to modify their physical, social, or digital environments through artifacts, nudges, or rituals. The next major assignment has students formally pitch a Nudge Unit, as if to a healthcare CEO, and receive feedback on their technique. A Nudge Unit provides system-wide expertise in identifying opportunities, designing, creating demand for, implementing, managing, and evaluating nudge projects to improve healthcare (20). After 6 months they must complete a halftime reflection presentation on major takeaways for how to leverage course tools, processes, and strategies in their professional work. With the remaining half of the course, the focus on nudging and branding shifts to storytelling and differentiating signals from noise. The following assignment divides cohorts into four groups, each assigned a case study of where Agile Innovation, Implementation, or Diffusion has been successful within the healthcare delivery system. Each month following the project introduction, a team must present their case studies to the cohort with action-oriented advice and an innovative delivery method, utilizing storytelling or gamification. In addition to these assignments, optional out-of-class chances for practice have included Innovation Forums, engineering a network of program alum, or Agile Implementation consulting. In the final months, students must draft their “why” statements, used when creating demand, and run sprints employing trial and error methods to improve (see Supplementary Appendix 2). The program ends with a final project, where members each submit a playbook and complete a final presentation. A playbook is a word document summarizing all actionable notes from the course. Usually, playbooks include information on Agile Science (behavioral economics, complex adaptive systems, network science), Agile mindset (psychological safety, feedback, sprints), Agile cycle (planning, reflecting, adjusting, sprinting), the 3 Agile processes (Innovation, Implementation, Diffusion), Agile tools and strategies (storytelling, nudging, branding, pitching). During the last weekend residency, each learner must present their personal journey, reflect on takeaways from the course, and create a blueprint to operationalize the content. Presentations can range anywhere from 15 min to 1 h and feedback is given immediately after.

### IIS Certificate Program curriculum

The curriculum is broken down into 6 primary sections: Innovation and Implementation Science I; Health Outcomes and Evaluation in Implementation Science; Innovation and Implementation Science II; Leading Change, Teams, and Projects; Practicum in Innovation and Implementation Science I; and Practicum in Innovation and Implementation Science II (19). The primary topics covered are behavioral economics, complex adaptive systems theory, network science, nudge theory, storytelling, branding, Agile Innovation, Agile Implementation, and Agile Diffusion.

Agile Science leverages insights from behavioral economics, complex adaptive systems theory, and network sciences to understand, predict, and steer the behavior of an individual or a social organization (see Supplementary Appendix 2). Behavioral economics studies the influence of psychological, cognitive, emotional, cultural, and social factors on human decision-making (18). In behavioral economics, System 1 cognitive processing is automatic, quick, and relies on biases, where System 2 cognitive processing is deliberate, logical, and less dominant (21). Errors in System 1 processing produce cognitive biases (21). Designed by System 2 processing, nudges account for System 1 errors using innovative ways to drive human behavior toward best practice (22). Nudges architect a physical, social, or digital environment to facilitate certain behaviors without forbidding choice and without monetary value (22). A complex adaptive system (CAS) is a network of diverse semi-autonomous subsystems, called agents, that traces conditional interactions between individuals and their environments, which drives the network (23). The healthcare delivery system is one such example, explaining why current efforts at change are often futile in altering emergent behavior. Network science builds on CAS, where individual persons are nodes and highly connected nodes are hubs (28). The conditional information or resource exchanges between nodes are called links (28). Nodes are clustered in social communities which are connected by bridges (28). A complex adaptive human network also has a semi-permeable boundary regulating information and energy flow (18, 24).

Constructed from the scientific foundation of behavioral economics, CAS, and network science, Agile processes for innovation, implementation, and diffusion provide steps to select, integrate, and scale evidence-based solutions within the constraints of healthcare delivery organizations (see Supplementary Appendix 2). All three processes require systemic demand, designate evaluation and termination plans, use sprinting to rapidly test and modify solutions, and have a minimally viable architecture for disseminating findings (18, 24).

### Data collection

Each graduate from the 2016–2021 cohorts was sent a personalized survey in 2022 with their name, email, and graduation year pre-filled out to prevent repeat responses. A reminder email was sent after 3 weeks and after 4 weeks following the initial email to those who had yet to complete the survey. All 42 questions in the survey were multiple choice to minimize survey fatigue, with the estimated time to complete the survey under 10 min. The questions were chosen based on the ability to measure the expansion of learned Agile tools, processes, and strategies more widely into spheres of influence. The survey was broken down into three sections: professional application, current competency as an Agile Change Conductor, and frequency of usage for tools, processes, and strategies obtained in the program. In the professional application section, graduates were asked to complete the Net Promotor Score question “On a scale of 0 to 10, how likely are you to recommend our certificate to a friend or colleague?” Net Promoter Scores are a standardized metric with national data available for how different economic sectors rank (25). In addition, the graduates were asked to rank 6 statements on a scale ranging from “(1) Strongly Disagree” to “(5) Strongly Agree” (see Supplementary Appendix 1). This is consistent with a Likert scale measuring the intensity of attitudes (26). The competencies section asked about the degree of mastery in various tools, processes, and strategies used by the Agile Change Conductor. Competencies are domain and context specific criteria that can be improved through training and deliberate practice, characterized by knowledge of theories and skill proficiency (27). The list of competencies was developed by Dr. Malaz Boustani, MD, MPH, and Dr. Jose Azar, MD, the Founding Directors of CHIIS, using goal-directed change management insight from Agile Science with a focus on innovation, implementation, and diffusion. Each item was measured between 0 and 10, with 0 indicating no understanding and 10 that of mastery (see Supplementary Appendix 1). In the final section, graduates were asked to rank how frequently they used various tools, processes, and strategies developed by the Graduate Certificate course, on a scale ranging from “(1) Never” to “(5) In my day-to-day life” (see Supplementary Appendix 1).

An additional database was created about graduates' professional advancements using public LinkedIn™ information and employer web pages. The data compiled consisted of current occupation, how long they had been in that position, the number of job updates following graduation, schooling updates since graduation, the number of followers if applicable, and any major accomplishments since program completion. No formal Certificate Program follow-ups had been conducted prior to this study. Hence, this served to track changes in professional development following the completion of the program. For ethical considerations, all information was anonymized following data acquisition and stored on a password-protected platform.

### Analysis

We used quantitative descriptive statistics and pre and post comparison statistics to assess the impact of the program. Given the numerical ranking of all 42 questions, overall averages and standard of deviations were computed for each question and each cohort. Pre and post training competency comparisons were computed for the 2020 and 2021 cohorts, a subsample of the study population, using 2-sample *t*-tests at a 95% confidence interval. ANOVA testing was used to identify any significant temporal differences between cohort years.

## Results

The first six cohorts included 25 physicians, nine nurses, 14 masters-level administrators, three social workers, and nine other clinically trained personnel. Participants for this study included all 60 past students of the program. Because the first cohort graduated in 2016, there were a total of six cohorts with varying amounts of participants in each, the most recent having graduated in August of 2021. Of the 60 alumni contacted, 52 completed the survey yielding an 87% response rate. Sixty percent of the graduates were female and 30% were of an under-represented minority (African American, Asian, Hispanic). Using the Net Promotor Scale, 36 participants (69%) were promoters of the program with a score of 9 or 10; one participant (2%) was considered a detractor with a score below 7; and finally, 15 participants (29%) were neutral with a score of 7 or 8. Thus, the Net Promoter Score was 67 (promoters minus detractors). Neutral and detractor participants came from every cohort and a diverse range of healthcare occupations.

In terms of the professional benefit of the program (see Table 1), graduates agreed that the certificate provided significant benefits for advancing their professional career [4.308 with a standard deviation (SD) of 0.612]; expanded their professional network (4.615, SD of 0.530); had a large impact on the effectiveness of their leadership (4.288, SD of 0.667), their change management (4.365, SD of 0.742), and their communication (4.392, SD of 0.666); and finally the graduates agreed that Agile Science has been applicable in their field (4.438, SD of 0.727).

**Table 1:** Survey results of the application of graduate certificate on career.

Questions^**^(scale: 1–5)^*^	**Average (*n* = 52)**	**St. dev (*n* = 52)**	**2021 average (*n* = 13)**	**2020 average (*n* = 6)**	**2019 average (*n* = 15)**	**2018 average (*n* = 4)**	**2017 average (*n* = 3)**	**2016 average (*n* = 11)**
The innovation and implementation certificate program advanced my professional career.	4.308	0.612	4.308	4.667	4.067	4.500	4.000	4.455
Agile Science has been applicable to my field of work.	4.538	0.727	4.769	4.667	4.333	4.500	4.333	4.545
The graduate certificate expanded my professional network.	4.615	0.530	4.462	4.500	4.667	4.750	4.333	4.818
The program had a large impact on the effectiveness of my leadership.	4.288	0.667	4.231	4.333	4.133	4.750	4.333	4.364
The program had a large impact on the effectiveness of my change management.	4.365	0.742	4.231	4.500	4.333	4.750	4.333	4.364
The program had a large impact on the effectiveness of my communication.	4.392	0.666	4.308	4.167	4.357	4.500	4.667	4.545

* Scale: (1) Strongly disagree (2) disagree (3) neutral (4) agree (5) strongly agree.

** No trend observed between cohorts (*p* = 0.06).

Table 2 summarizes the competencies of the graduates in being Agile Change Conductors using a scale from 0 to 10 where 10 is indicative of reaching mastery. The total average score for each of the 26 different competencies ranged from 5.37 (SD of 2.80) for writing grants or business proposals to 7.77 for self-awareness (SD of 1.96). There were no statistically significant differences between the 2016 and 2021 cohorts (*p* = 0.52). Existing competency data taken prior to course completion existed for the 2020–2022 cohorts (*n* = 34). There were no statistically significant differences between pre-completion scores over the 3 years (*p* = 0.99). Twenty-two out of the 26 competencies saw a statistically significant increase from pre to post training proficiency (*n* = 18). The exceptions were in writing papers, writing grants or business proposals, statistical analysis to pick up signal from noise, and traditional research methodology.

**Table 2:** Agile competency following course completion.

Agile competencies (scale: 0-10)^*^	Average score per question (*n* = 52)^**^	**Competency st. dev per question (*n* = 52)**
Questioning	7.54	1.379
Deep observing	7.67	1.723
Experimentation	7.33	1.723
Network for discovery	7.17	1.779
Network for resources	7.25	1.898
Associative thinking	7.65	1.494
Innovation in limited	7.19	1.858
Matching for innovative solutions	6.98	1.767
Writing papers	6.31	2.478
Writing grants or business proposal	5.37	2.801
Statistical analysis to pick up a signal	6.00	2.59
Traditional research methodology	6.25	2.375
Behavioral economics	7.23	1.946
Complex adaptive system theory	7.04	1.857
The steps of Agile innovation	7.20	1.855
The steps of Agile implementation	7.56	1.893
Agile analytics	6.27	2.357
Zoom-in/zoom-out	7.29	1.913
Storytelling	7.35	2.15
Nudging or choice architect	7.41	1.931
Leading >150 people toward a common goal	5.48	2.783
Building highly functional team	6.94	2.209
Social awareness	7.63	1.8
Self-awareness	7.77	1.957
Social intelligence	7.51	1.891
Branding	6.83	1.958

* Scale: no competency (0) (1) (2) (3) (4) (5) (6) (7) (8) (9) (10) Mastery.

** No trend observed between cohorts (*p* = 0.52).

Regarding the frequency of utilizing the tools, processes, and strategies obtained from the program (see Table 3), the average scores ranged from monthly to weekly use. Graduates claimed to use Agile processes (Innovation, Implementation, or Diffusion), storytelling, and nudging weekly. The majority of graduates deliberately brand themselves and review their playbooks monthly.

**Table 3:** Survey results on the frequency of usage for the tools, processes, and strategies obtained from the program.

Questions^**^(scale: 1–5)^*^	**Average (*n* = 52)**	**St. dev (*n* = 52)**	**2021 average (*n* = 13)**	**2020 average (*n* = 6)**	**2019 average (*n* = 15)**	**2018 average (*n* = 4)**	**2017 average (*n* = 3)**	**2016 average (*n* = 11)**
How often do you use Agile Innovation, Implementation, or Diffusion?	3.692	1.229	3.769	4.000	3.333	3.750	3.667	3.909
How regularly do you review your playbook or notes?	2.538	0.999	2.538	2.667	2.333	2.75	2.333	2.727
How often do you use storytelling?	3.731	1.122	3.615	3.833	3.800	3.000	3.667	4.000
How often do you try to implement Nudges?	3.500	1.000	3.308	3.500	3.467	3.500	3.667	3.727
How often do you make an effort to deliberately brand yourself to others?	3.212	1.194	3.231	3.500	3.067	2.500	3.000	3.545

* Scale: (1) Never, (2) A few times a year, (3) A few times a month, (4) A few times a week, (5) In my day-to-day life.

** No trend observed between cohorts (*p* = 0.71).

Based on information from publicly available databases, 65% of graduates had a promotion and/or have since pursued an additional degree or professional training. The average number of followers was 846 based on available LinkedIn™ accounts. The average number of years in their current position was 4.11 years (SD of 4.73). For career advancements, the average number increased from 0.25 one year out to two changes 6 years out (see Table 4).

**Table 4:** Average career advances for graduates per cohort.

**Year graduatd (*n* = 59)**	**Average # of years in current position**	**Average # of linkedin followers**	**Average # of job promotions/changes since graduating**	**Average # of education updates since graduating**
2021 (*n* = 13)	4.73	1,456	0.25	0.42
2020 (*n* = 7)	6.30	140	0.50	0.00
2019 (*n* = 16)	3.90	610	0.69	0.13
2018 (*n* = 5)	4.46	942	1.60	1.60
2017 (*n* = 3)	4.50	406	1.00	0.67
2016 (*n* = 15)	2.96	925	1.93	0.20
Average for all graduates	4.11	846	1.00	0.35

## Discussion

We found that the IIS Certificate Program has impacted graduates in terms of their professional careers, network expansion, change management skills, communication, and leadership. The program was also successful in building a network of Agile Change Conductors who are strong promoters of the program and competent in implementing and diffusing evidence-based care in their local complex adaptive healthcare delivery organizations. This indicates the Certificate Program is an effective way to create Agile Change Conductors capable of designing, implementing, and diffusing evidence-based healthcare solutions.

We used the Net Promoter score to measure the graduates' attitudes toward the program. A score above 70 is considered excellent (28). The program had a Net Promoter score of 67 indicating overall satisfaction with having taken the Certificate Program. Coupling this score with the high impact of the certificate on professional careers, the high competency of the graduates, and their high use of the tools, processes, and strategies obtained *via* the program exemplifies the success of the program in building an integrated network of Agile Change Conductors.

The frequency of usage section in the survey helps monitor the influence of the program on the daily activities of graduates. While the scores had more variation between graduates, the results were still overwhelmingly positive. No question had an average score below 2.5, which translates onto monthly use. Weekly use was the most common response for using Agile Processes (Implementation, Innovation, and Diffusion), storytelling, and nudging, demonstrating a high amount of practice. The integration of course material into the habitual lives of the graduates is a testament to sustained impact that goes beyond knowledge acquisition.

Majority of graduates did not have an active LinkedIn™ account prior to enrollment in the Certificate Program. Students without accounts were strongly encouraged to create a profile for the purposes of branding and networking for resources and discovery. Therefore, 92% of the graduates having an active account with an average of over 800 connections reasonably demonstrates the Certificate Program expanded professional networks. From profiles, there was professional advancement of most members (65%), in line with most graduates agreeing that the program benefited them professionally through position changes, continued education advances, and/or achievements.

When looking at the success of the program in creating well-rounded Agile Change Conductors equipped with transferable change management skills, the proficiency test indicates graduates are competent and nearing mastery. For each of the 26 competencies, the data were skewed left with averages showing nearly all members were more competent than not. The standardized increase in competency for nearly all skills of an Agile Change Conductor in a subsample of the study population is indicative of some causal benefit from the program. Proficiency did not appear to decline, given no variation between the 2016 cohort surveyed 6 years following graduation and the 2021 cohort surveyed 1 year following graduation. There was no decline in competency for either the 2020 or 2021 cohorts despite the shift to fully virtual learning. These results indicate satisfactory improvements in each of the intended competencies, highlighting the Graduate Certificate Program has thus far met its professed goals of training members in innovation and implementation science.

The University of California San Francisco Certificate Program in Implementation Science has similarly seen moderate to high confidence in 12 self-assessed competencies (*n* = 54, 2008–2017), coming out in strong support of the benefits of graduate-level training in implementation science career development (29). These results match the current study's moderate to high levels of mastery for all competencies. The University of South Florida offers an Implementation Science Graduate Certificate Program through The Institute for Translational Research Education in Adolescent Drug Abuse (30). Using semi-constructed interviews (*n* = 58), the program has been found to create a robust and transferable skillset for researchers and healthcare practitioners (30). Despite the success of the IU-CHIIS Graduate Certificate Program and similar ones across the nation, there is still demand for an increases in the number of advanced education programs in innovation, implementation, and diffusion sciences to systematically increase the use of evidence-based health practices (27). Continued expansion of Agile Diffusion and improvements in developing signal-noise detection, leadership, and branding skills will prove crucial for steering information and behavior change in widespread complex adaptive human networks (24). Disseminating the IU-CHIIS curriculum seeks to provide a framework for future educational programs capable of creating Agile Change Conductors within the healthcare delivery system.

Competencies with the lowest averages were the ability to write grants or business proposals and leading 150 people toward a goal, both of which have larger amounts of variability likely due to how applicable these specific competencies are to some professions compared to others. Areas without significant improvements also included writing papers and traditional research methodology. Selective bootcamps on Agile Science specified for professionals in healthcare delivery business operations, implementation and innovation science research, or healthcare administrative leadership are advised for directed aptitude development in drafting business proposals, writing papers or research methodology, and grant writing or leading 150 people toward a goal, respectively. Signal-noise detection was another Agile competency with space for improvement. Continued research at IU-CHIIS on Agile analytics, with better ways to distinguish signals from noise will help address such gaps. The development and addition of an immersive simulation-based portion of the curriculum in the near future will likely provide further opportunities to develop mastery of the core principles of Agile Science while providing objective data on the ability of graduates to optimally capitalize on their training. A qualitative study interviewing past graduates will be needed to provide direct feedback and recommendations for future years.

There remain several limitations to this study. First, since the primary source of data was a survey with subjective responses, this provides room for bias in the responses. Responses may be less critical of the program. The survey was de-identified but not anonymous due to the necessity of knowing cohort years and to help monitor which alumni had yet to complete the survey. However, data was analyzed after being de-identified, blinding researchers to identifiable information. Furthermore, even though the response rate was high (87%), the survey lacked eight responses to make the data complete. It is therefore possible the data is not completely representative of all past cohorts, especially early cohorts with lesser response rates. A significant limitation of this study is the lack of baseline competency data for cohorts 2016–2019, greatly reducing the comparative aspect of this study. Majority of conclusions are made using descriptive statistics. While the absence of variation in competency across cohorts may help generalize the 2020–2021 pre-post training findings more broadly, the small sample size limits direct causal attributions. Additionally, there are likely large parts of careers not posted on LinkedIn™. It is difficult to know the extent to which career advances seen online were due to the program, as is also the case with follower counts. Despite these limitations, the data overwhelmingly points to a beneficial impact of the program.

In conclusion, the Certificate Program in Health Innovation and Implementation Sciences has been successful in creating Agile Change Conductors who have been able to act upon the set of learned tools, processes, and strategies to transform the healthcare delivery system.

## Data availability statement

The original contributions presented in the study are included in the article/Supplementary material, further inquiries can be directed to the corresponding author.

## Ethics statement

The studies involving human participants were reviewed and approved by Indiana University Institutional Review Board (#15824). The patients/participants provided their written informed consent to participate in this study.

## Author contributions

JM: primary author of the paper, designed, conducted, and analyzed the study, drafted and edited the paper, and developed all supplementary materials. MA: developed idea and initiative for paper and edited paper and survey throughout the process. AO'B, TB, RA, and DS: editors for the paper and made significant contributions to the rhetoric and outstanding ideas presented in the final version. MB: principle investigator, helped design, conducted, analyzed, and wrote about the study. All authors contributed to the article and approved the submitted version.
