# Supplementary material for: Becoming an Agile Change Conductor
Source: Front Public Health. 2022 Dec 14;10:1044702. doi: 10.3389/fpubh.2022.1044702 (PMC9794851; doi:10.3389/fpubh.2022.1044702)

**Supplemental Appendix 1: Survey Questions**

Pre-Filled In: Name, Email, Year of Graduation

Professional Application

Net Promoter Score: On a scale from 0-10, how likely are you to recommend the CHIIS Certificate Program to a friend or colleague?

0) 1) 2) 3) 4) 5) 6) 7) 8) 9) 10)

*On a scale from 1 to 5 rate how much you agree with the following statements…*

The innovation and implementation certificate program advanced my professional career.

1) Strongly Disagree

2) Disagree

3) Neutral

4) Agree

5) Strongly Agree

Agile Science has been applicable to my field of work.

**Agile Science leverages insight from behavioral economics, complexity science, network science to understand, predict, and steer the behavior of a human individual and the behavior of a social human organization.

1) Strongly Disagree

2) Disagree

3) Neutral

4) Agree

5) Strongly Agree

The graduate certificate expanded my professional network.

1) Strongly Disagree

2) Disagree

3) Neutral

4) Agree

5) Strongly Agree

The program had a large impact on the effectiveness of my leadership.

1) Strongly Disagree

2) Disagree

3) Neutral

4) Agree

5) Strongly Agree

The program had a large impact on the effectiveness of my change management.

1) Strongly Disagree

2) Disagree

3) Neutral

4) Agree

5) Strongly Agree

The program had a large impact on the effectiveness of my communication.

1) Strongly Disagree

2) Disagree

3) Neutral

4) Agree

5) Strongly Agree

Current Competency as an Agile Change Conductor

This section will ask you how competent you feel you are currently regarding different skills, qualities, and theories from the graduate certificate course.

*Please complete the below items using a scale from 0 to 10. There will be 4 sections total.*

*Section 1: Innovation*

Questioning

No Competency 0) 1) 2) 3) 4) 5) 6) 7) 8) 9) 10) Mastery

Deep Observation

No Competency 0) 1) 2) 3) 4) 5) 6) 7) 8) 9) 10) Mastery

Experimentation

No Competency 0) 1) 2) 3) 4) 5) 6) 7) 8) 9) 10) Mastery

Networking for Discovery

No Competency 0) 1) 2) 3) 4) 5) 6) 7) 8) 9) 10) Mastery

Networking for Resources

No Competency 0) 1) 2) 3) 4) 5) 6) 7) 8) 9) 10) Mastery

Associative Thinking

No Competency 0) 1) 2) 3) 4) 5) 6) 7) 8) 9) 10) Mastery

Innovation in a Limited Environment

No Competency 0) 1) 2) 3) 4) 5) 6) 7) 8) 9) 10) Mastery

Matching for Innovative Solutions

No Competency 0) 1) 2) 3) 4) 5) 6) 7) 8) 9) 10) Mastery

*Section 2: Scholar 1.0 Skills*

Writing Papers

No Competency 0) 1) 2) 3) 4) 5) 6) 7) 8) 9) 10) Mastery

Writing Grants or Business Proposals

No Competency 0) 1) 2) 3) 4) 5) 6) 7) 8) 9) 10) Mastery

Statistical Analysis (to Pick up Signal from Noise)

No Competency 0) 1) 2) 3) 4) 5) 6) 7) 8) 9) 10) Mastery

Traditional Research Methodology

No Competency 0) 1) 2) 3) 4) 5) 6) 7) 8) 9) 10) Mastery

*Section 3: Scholar 2.0 Skills*

Behavioral Economics

No Competency 0) 1) 2) 3) 4) 5) 6) 7) 8) 9) 10) Mastery

Complex Adaptive Systems and Network Science

No Competency 0) 1) 2) 3) 4) 5) 6) 7) 8) 9) 10) Mastery

The Steps of Agile Innovation

No Competency 0) 1) 2) 3) 4) 5) 6) 7) 8) 9) 10) Mastery

The Steps of Agile Implementation

No Competency 0) 1) 2) 3) 4) 5) 6) 7) 8) 9) 10) Mastery

The Steps of Agile Diffusion

No Competency 0) 1) 2) 3) 4) 5) 6) 7) 8) 9) 10) Mastery

*Section 4: Leadership*

Zoom-in/Zoom-out

No Competency 0) 1) 2) 3) 4) 5) 6) 7) 8) 9) 10) Mastery

Storytelling

No Competency 0) 1) 2) 3) 4) 5) 6) 7) 8) 9) 10) Mastery

Nudging or Choice Architecting

No Competency 0) 1) 2) 3) 4) 5) 6) 7) 8) 9) 10) Mastery

Leading more than 150 People Towards a Common Goal

No Competency 0) 1) 2) 3) 4) 5) 6) 7) 8) 9) 10) Mastery

Building a Highly Functional Team

No Competency 0) 1) 2) 3) 4) 5) 6) 7) 8) 9) 10) Mastery

Social Awareness

No Competency 0) 1) 2) 3) 4) 5) 6) 7) 8) 9) 10) Mastery

Self Awareness Social Intelligence

No Competency 0) 1) 2) 3) 4) 5) 6) 7) 8) 9) 10) Mastery

Branding

No Competency 0) 1) 2) 3) 4) 5) 6) 7) 8) 9) 10) Mastery

Frequency of Usage of Tools, Processes, and Strategies

How often do you use Agile Innovation, Implementation, or Diffusion?

1) Never

2) A few times a year

3) A few times a month

4) A few times a week

5) In my day-to-day life

How regularly do you review your playbook or notes?

1) Never

2) A few times a year

3) A few times a month

4) A few times a week

5) In my day-to-day life

How often do you use storytelling?

1) Never

2) A few times a year

3) A few times a month

4) A few times a week

5) In my day-to-day life

How often do you try to implement Nudges?

1) Never

2) A few times a year

3) A few times a month

4) A few times a week

5) In my day-to-day life

How often do you make an effort to deliberately brand yourself to others?

1) Never

2) A few times a year

3) A few times a month

4) A few times a week

5) In my day-to-day life

**Supplemental Appendix 2: Supplementary Figures**


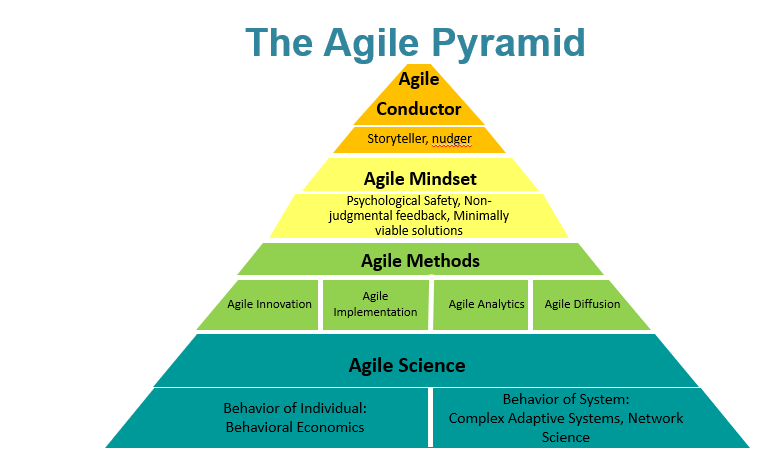


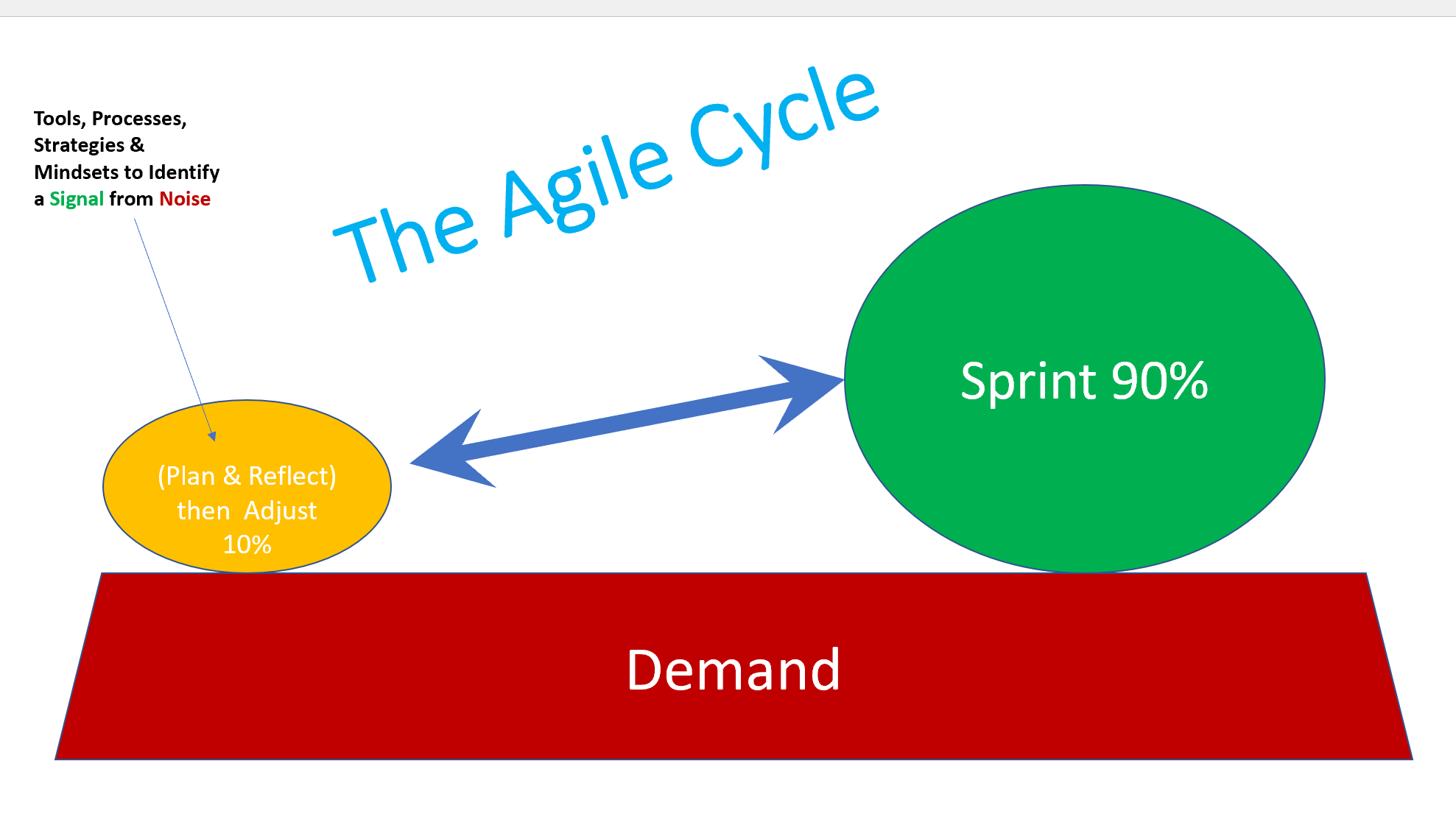


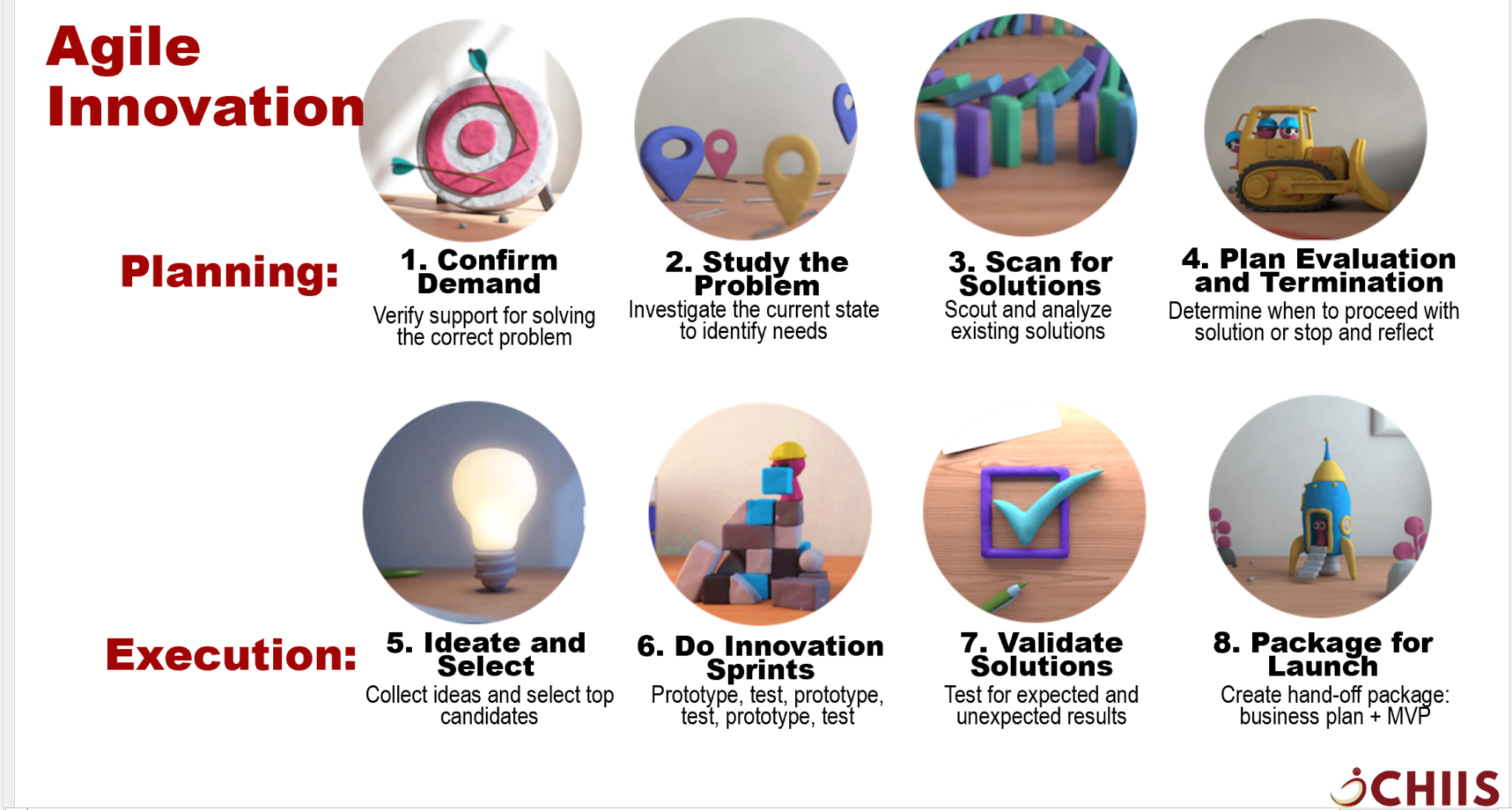


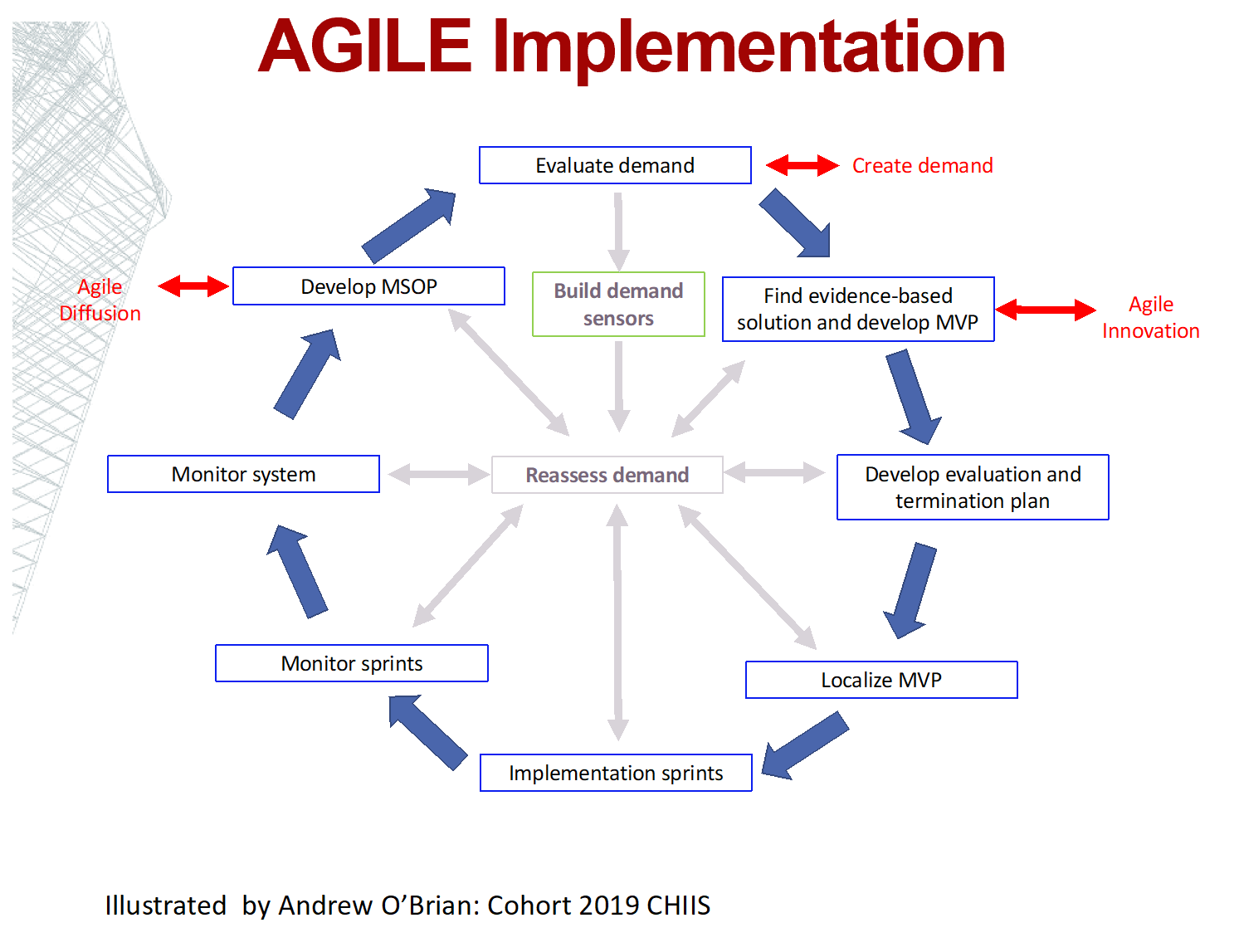


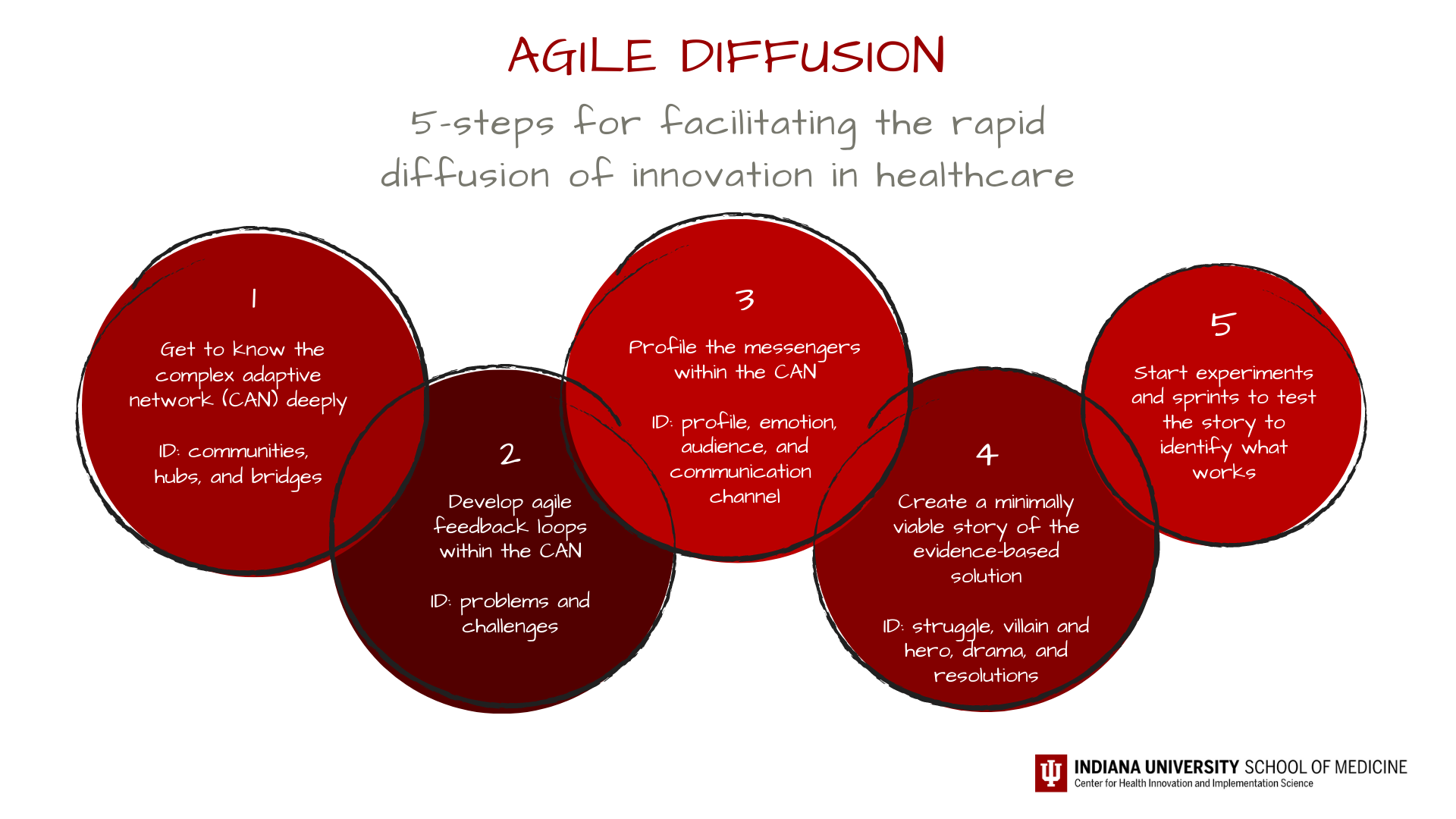


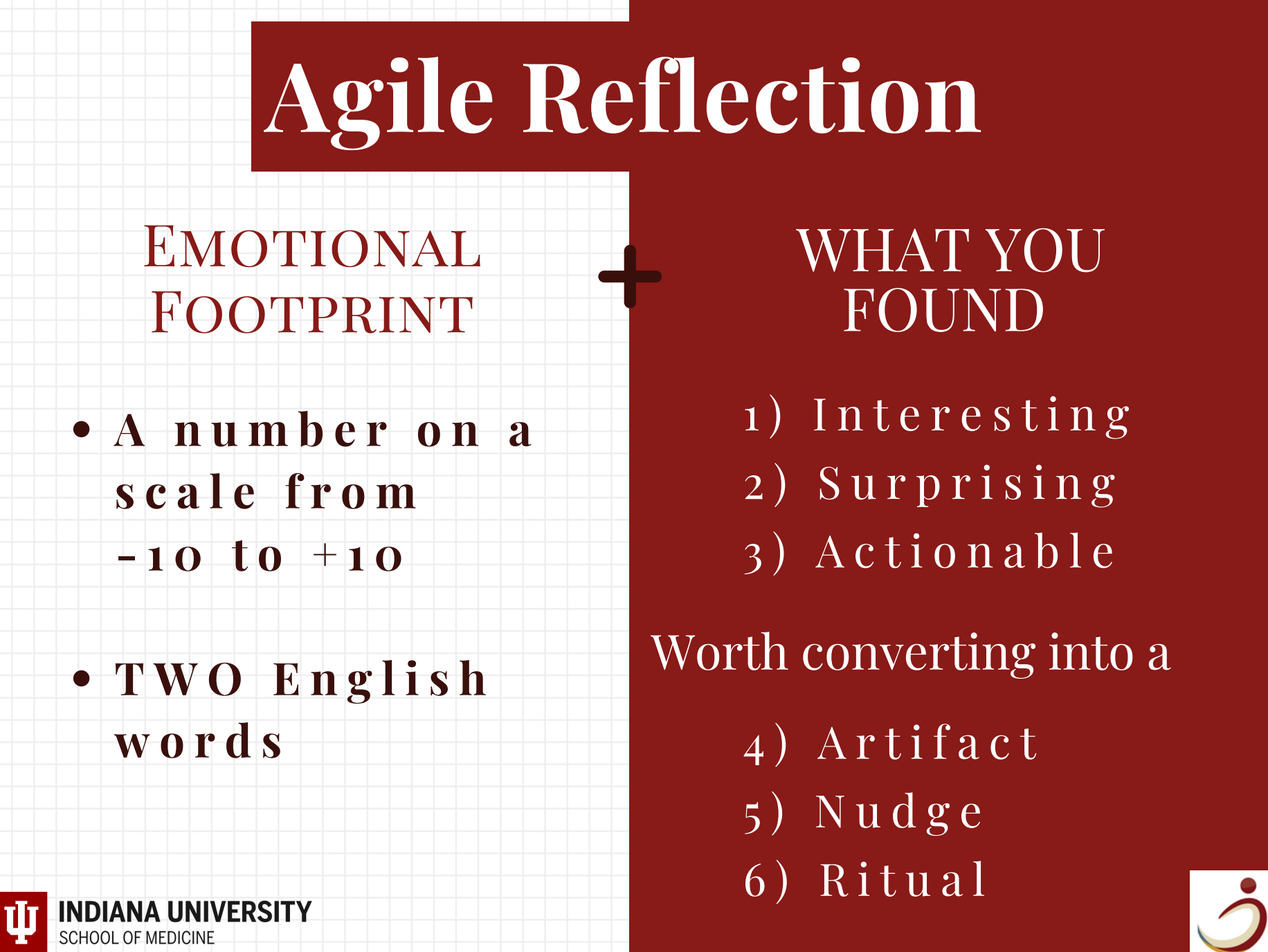


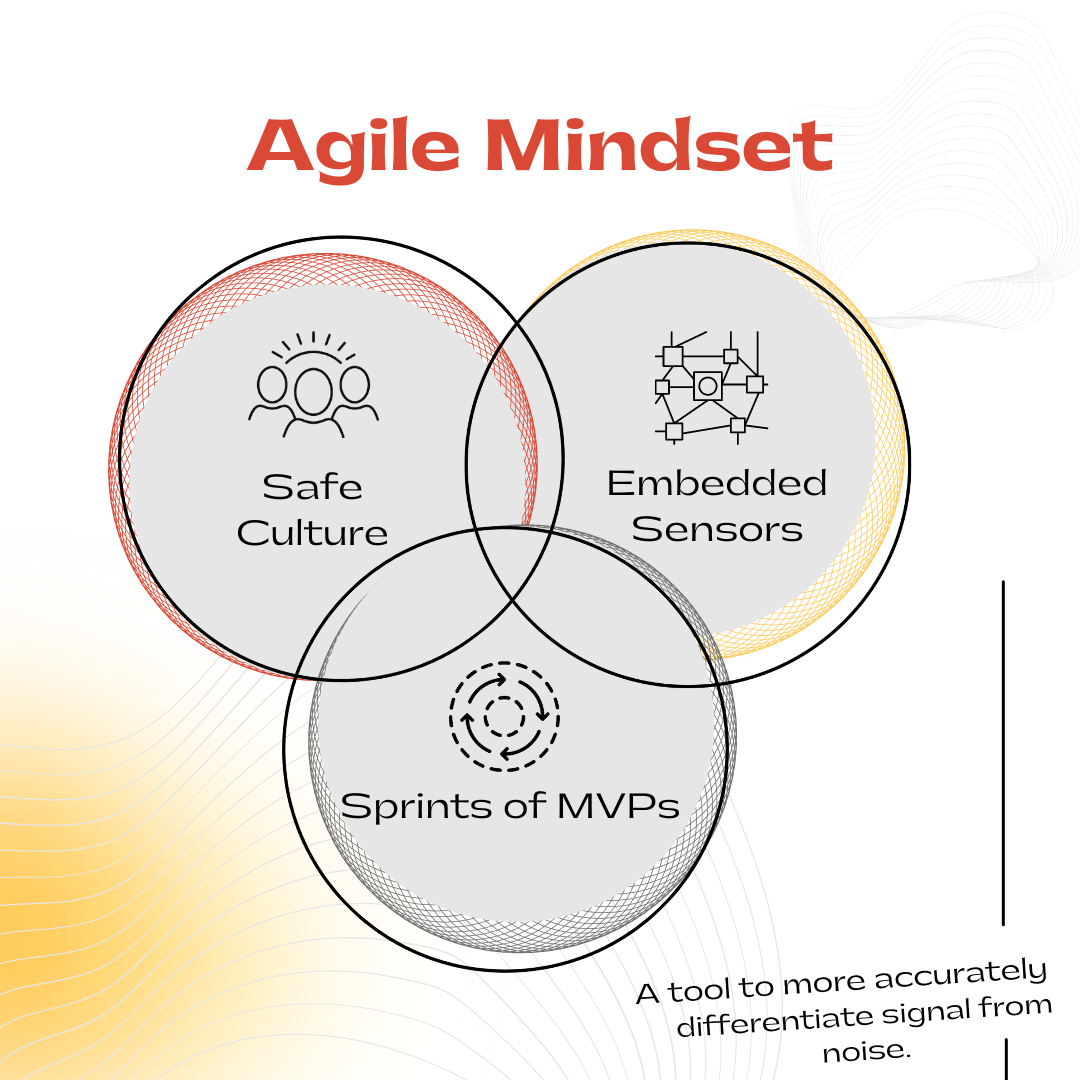

Supplement: Supplementary file 1 [file Table_1.DOCX]
